# Emotion and Wellness Profiles of Herbal Drinks Measured Using Different Questionnaire Designs

**DOI:** 10.3390/foods11030348

**Published:** 2022-01-26

**Authors:** Pannapa Hanmontree, Witoon Prinyawiwatkul, Amporn Sae-Eaw

**Affiliations:** 1 Department of Food Technology, Faculty of Technology, Khon Kaen University, Khon Kaen 40002, Thailand; pannapa@kkumail.com; 2 School of Nutrition and Food Sciences, Louisiana State University, Agricultural Center, Baton Rouge, LA 70803, USA; wprinya@lsu.edu

**Keywords:** emotion, wellness, herbal drink, product discrimination, questionnaire designs

## Abstract

The emotion and wellness profiles of herbal drinks were assessed using six different questionnaire designs. The questionnaire designs were constructed from two formats of questionnaire items, including words and sentences, and three types of measuring scales, including a rating scale (5-point intensity; 1 = ‘not at all’, 5 = ‘extremely’), a checklist scale (check-all-that-apply, CATA), and a combination of CATA and rating scales (rate-all-that-apply, RATA; 5-point intensity; 1 = ‘slightly’, 5 = ‘extremely’). The 39 emotional terms of the EsSense Profile^®^ and the 45 wellness terms of the WellSense Profile^TM^ were translated into Thai, then screened for relevance to herbal drinks. The seven positive emotional terms (active, energetic, good, happy, polite, satisfied, and warm), three negative emotional terms (bored, disgusted, and worried), and five wellness terms (comforted, healthy, invigorated, relaxed, and refreshed) were selected and included in the questionnaire. A central location test was performed to determine the emotion and wellness profiles of five herbal drinks: roselle (*Hibiscus sabdariffa*) drink, chrysanthemum (*Chrysanthemum morifolium Ramat*) drink, ginger (*Zingiber officinale)* drink, Jubliang (a mixture of eight herbs) drink, and Krachai Dam (*Kaempferia parviflora)* drink. For herbal drinks, measuring emotion and wellness with a questionnaire using full sentences did not show increased benefit over questionnaires using words alone. All three measuring methods—a rating scale, CATA, and RATA—produced similar emotion and wellness profiles. However, each method has different advantages and limitations, which researchers should carefully consider.

## 1. Introduction

In the last decade, emotional responses have been more frequently used in consumer research and food-product development because the use of liking data alone may not effectively predict product success in the market. Collecting emotional responses that offer insight into consumer experience can provide additional useful information for product development [1]. Several findings have noted that emotions influence consumers’ eating behavior and decision-making processes [2,3,4]. Understanding the emotions elicited by their products could be beneficial for food industries when considering packaging design, branding, and advertising [2].

Several methods for measuring emotions associated with foods have been developed and reported, such as EsSense Profile^®^ [5], consumer-defined check-all-that-apply (CD-CATA) [6], EmoSemio [7], and EmoSensory^®^ Wheel [8]. Furthermore, the health and wellness perception of food products has become an important aspect of consumers’ purchasing decisions. The WellSense Profile^TM^, a questionnaire measuring wellness associated with foods from the consumer perspective, was recently developed [9] and was adapted in recent research to measure organic food-related wellbeing [10] in relation to the Krachai Dam drink [11]. Accordingly, the emotion and wellness responses of consumers should be considered in food-product development. Although the EsSense profile^®^ and the WellSense Profile^TM^ methods have been widely used, the application of these methods in in countries with different cultures and languages should be appropriately performed. For this reason, cross-cultural studies of emotion elicited by food have gained attention [12,13,14]. However, research investigating the impact of questionnaire design and measurement scales on emotion and wellness profiles is scarce. In Thailand, methods for measuring emotional response elicited by food products have not been widely applied, so more research is required.

Herbs have been recognized as having health benefits [15]. Herbs and their extracts contain different bioactive compounds that can provide therapeutic effects, such as reducing cardiovascular problems, enhancing immune functions, and preventing cancer [16]. In Thailand, herbs have been used as medicine and food for a long time. Herbal drinks are becoming more popular, especially among health-conscious consumers, since these beverages are prepared with natural ingredients. The results obtained in a study by Jabeur et al. [17] highlighted the potential of *Hibiscus sabdariffa* L. (Hs), also known as roselle, as a source of bioactive and natural coloring ingredients destined for food and pharmaceutical industries. Roselle extracts showed antibacterial, antioxidant, anticholesterol, antidiabetic and antihypertensive effects, among others [18]. In recent years, the flowers of medicinal chrysanthemum (*Chrysanthemum morifolium Ramat*) were shown to act as common materials in functional and healthy tea or beverages due to their unique flavor, color, and health benefits, such as detoxification, improving liver function, decreasing inflammation, and improving eyesight [19]. Ginger (*Zingiber officinale*) rhizomes are commonly used in foods and beverages for their characteristic pungency and piquant flavor and have exhibited various pharmacological effects, such as antioxidant, anti-inflammatory, gastroprotective, antibacterial, and antidiabetic properties [20]. Jubliang is a mixture of eight herbs, namely *Bombax ceiba* L., *Chrysanthemum morifolium*, *Imperata cylindrical* (L.) P. Beauv., *Lophatherum gracile* Brogn., *Nelumbo nucifera* Gaertn, *Oroxylum indicum* L., *Pragmites communis* Trin, and *Prunella vulgaris*. Jubliang extract is a good source of water-soluble antioxidants, phenolic compounds, and antimutagens [21]. *Kaempferia parviflora*, or Krachai Dam (KD), is a Thai herb that belongs to the *Zingiberaceae* family. Its rhizomes have been reputed to have beneficial medicinal effects, owing to its major methoxyflavones, including anticancer, cardioprotective, neuroprotective, antioxidative, antimicrobial, and transdermal-permeable activity [22].

There are many kinds of commercial herbal drinks in Thailand, some of which are very popular; others are not popular, even though they have health-promotion benefits. There are many factors that have influence consumer perception and buying decisions. An important factor is the emotion and wellness responses of consumers. Additionally, different questionnaire designs may influence the results of emotion and wellness assessments. We hypothesized that using a questionnaire designed with full sentences and a rating-scale measurement method would have a greater influence on emotion and wellness profiles than that with words and a check-all-that-apply (CATA) measurement method. Therefore, in this research, we aimed to examine and compare the emotion and wellness responses associated with herbal drinks using questionnaires constructed according to two formats—words vs. full sentences—and three types of measurement methods, including a rating scale, a checklist scale (CATA), and a combination of the two scales (i.e., RATA).

## 2. Materials and Methods

### 2.1. Herbal Drink Samples

Five herbal drink samples commercially available in Thailand were used, including roselle drink, chrysanthemum drink, ginger drink, Jubliang drink, and Krachai Dam drink (Table 1). Roselle drink, chrysanthemum drink, Jubliang drink, and Krachai Dam drink were ready-to-drink products. After purchased, they were kept at 4 °C until used for consumer testing. Samples were presented to the participants in random 3-digit labelled cups at approximately 10 °C, except ginger drink, which was served at approximately 70 °C. The ginger drink was prepared immediately prior to sampling by adding 18 g of instant ginger powder to 150 mL of hot water and kept in an insulated bottle. 

### 2.2. Screening of Emotion and Wellness Terms for Relevance to Herbal Drinks

The research protocol concerned with the use of human subjects was approved by the Khon Kaen University Ethics Committee for Human Research (KKUEC), Thailand (approval code, HE611230). Emotion and wellness terms were selected from the EsSence Profile^®^ [5] and WellSense Profile^TM^ tests [9]. All terms were translated into Thai by a group of experienced linguists (*n* = 3) and back-translated into English by another experienced group (*n* = 3) for meaning validation (Table 2). Thai emotion and wellness terms relevant to five herbal drinks (Roselle drink, Chrysanthemum drink, Ginger drink, Jubliang drink, and Krachai Dam drink) were screened using online CATA questionnaires. Thai consumers (*n* = 1678; 43.1% males and 56.9% females) who regularly consume herbal drinks participated voluntarily. Participants were asked to select the emotion and wellness terms that they associate with when thinking about herbal drinks that they had consumed. Terms with at least 20% frequency of use were recommended as a criterion [2,5,23,24]. However, terms with least 15% frequency of use were adopted for development of the EmoSensory^®^ Wheel, while negative emotion terms with a lower frequency of use (≤10%) were used to obtain a balance of positive and negative terms [8]. For our current research, terms with at least 18% frequency of use were used. First, the selected terms from all of five herbal drinks were collected. Then, the duplicated terms were removed before inclusion in the questionnaire. Based on the results, seven positive emotion terms (active, energetic, good, happy, polite, satisfied, and warm) and five wellness terms (comforted, healthy, invigorated, relaxed, and refreshed) were selected. To control the balance on both of positive and negative terms, three negative emotion terms (bored, disgusted, and worried) were added to the questionnaire (Table 3). Therefore, a total of 15 emotion and wellness terms were included in the questionnaire to measure the consumer responses elicited by the five herbal drinks.

### 2.3. Testing the Test Designs for Measurement of Emotion and Wellness Responses Elicited by Herbal Drinks

The proposed test designs for measurement of emotion and wellness responses were composed of 2 factors. Factor 1 was composed of two formats of questionnaire items, including words and full sentences (see the attached questionnaire file). The sentence format was used to reduce ambiguity within the questionnaire because including a context increases understanding of the specified emotion and wellness state. The full sentences were created using selected emotion and wellness terms, in consultation with a focus group of consumers (*n* = 29, average age = 45.76 ± 7.83 years old) (adapted from [7]). Factor 2 was composed of three types of measuring scales, including a rating scale (5-point intensity; 1 = ‘not at all’, 5 = ‘extremely’), a checklist scale (CATA), and a combination (rate-all-that-apply, RATA; 5-point intensity; 1 = ‘slightly’, 5 = ‘extremely’). Thus, the questionnaire designs comprised six test forms (Table 4).

The impact of the test designs (Test 1 to Test 6) was evaluated between 15 and 30 June 2018. Consumers were recruited by an accidental sampling method. According to Meilgaard et al. [25], 50–300 responses should be collected per location for the central location test (CLT). In this study, about 200 Thai consumers were recruited for each test design (a total of 1252; 49.3% males and 50.7% females) from 4 regions of Thailand (Table 4), with almost equal proportions of four age groups participating in the CLT. To be selected, consumers were required to be familiar with and frequently consumers of herbal drinks. Each consumer was randomly assigned to one of the six test designs. Five herbal drinks were coded with random 3-digit numbers. Each herbal drink sample (20 mL) was poured into a clear plastic cup and served under the corresponding condition/temperature described in Table 1. All five samples were served in a balanced order. All consumers received five herbal drink samples for tasting, along with water to cleanse their palates between samples. First, they were asked to taste and rate their overall liking of each sample using a 9-point hedonic scale (1 = extremely dislike, 9 = extremely like). Secondly, they indicated their emotion and wellness responses to each sample using the scales, as described above. Consumers were seated separately and were instructed not to interact with other participants.

### 2.4. Statistical Analysis

Analysis of variance (ANOVA) and Duncan’s multiple range test (DMRT) were performed on the overall liking scores to determine significant overall and pairwise differences among the five herbal drinks and six test designs.

For rating scores, the mean score of each emotion and wellness term was calculated from consumer responses. For CATA data, a frequency count was calculated by counting the number of consumers who chose the terms for each sample. RATA data were analyzed using one of two approaches [26]: frequency of selection (RATA frequency) or weighted frequency of selection (RATA score). RATA frequency was counted from the number of consumers who chose the terms for each sample. RATA scores were calculated by summing up the scores that the consumers rated for each selected term (0–5 scoring; 0 = ‘not selected’, 1 = ‘slightly’, and 5 = ‘extremely’) [27] and dividing by the number of participating consumers.

Both rating and RATA scores (when treated as a continuous outcome) were analyzed using ANOVA to determine the emotion and wellness terms with significant differences among the five herbal drinks. Next, the significant terms were analyzed using DMRT for multiple comparisons [28]. Principal component analysis (PCA) was performed to identify the emotion and wellness terms that contributed to overall differences among the herbal drinks and to classify the products based on emotion and wellness responses. Overall product differences were determined using multivariate analysis of variance (MANOVA) based on emotion and wellness responses, followed by discriminant analysis (DA) to identify the important terms of group discrimination.

CATA and RATA frequency counts were analyzed following the recommendation of Meyners et al. (2016) [27]. Cochran’s Q test was carried out to determine the differences among herbal drink samples for each emotion or wellness response, and the sign test was used for pairwise comparison. Correspondence analysis (CA) based on Chi-square distances was used to identify the emotion and wellness terms that correlated to overall differences among the sampled herbal drinks. DA was performed to identify the important terms of group discrimination.

All statistical analyses were performed at 5% significance level using the SPSS 19 software (SPSS Inc., Chicago, IL, USA), except DA, which was carried out using XLSTAT^®^ Basic version 2019 software (Addinsoft; New York, NY, USA).

## 3. Results and Discussion

### 3.1. Overall Liking of Herbal Drink Samples

Regarding the overall liking scores of the five herbal drinks from six test designs, the data were analyzed by ANOVA and DMRT (Table 5). In each test, significant differences in overall liking were observed (*p* < 0.05) among the five herbal drinks, indicating that consumers liked the five herbal drinks to varying degrees. In general, roselle and chrysanthemum drinks were similarly well-liked (7.11–7.49) and were found to be more acceptable than the three other herbal drinks (6.48–6.87 for ginger and Jubliang; 6.62–7.06 for Krachai Dam). For overall liking evaluation, different groups of consumers participated in each test design. Homogeneity of hedonic perception should be determined before comparing the different questionnaire designs. When comparing the overall liking score of each herbal drink among different consumers from the six test-design groups, no significant differences were found. This implies that the consumer population in this study was homogenous in terms of hedonic perception of the test samples. Thus, the subsequent emotion and wellness results of the six test designs could be compared since overall liking and emotion terms may be correlated, depending on product, product category, demographics, and psychographics [5].

### 3.2. Emotion and Wellness Profiles of Herbal Drinks from Different Test Designs

As seen from Table 6 and Table 7, the type of herbal drink significantly affected most emotion (except “good”, “happy”, “bored”, “worried”, and “disgusted” in some cases) and all wellness responses of consumers. Generally, negative emotion responses were rated lower than positive emotion and wellness responses. These findings confirm and extend the positive asymmetry of emotions. According to the theory of positive asymmetry, previous research findings reported that consumers experience primarily positive emotions in response to food and food names [29,30,31]. In the case of beverages, Calve-Porral et al. [32] found positive asymmetry of emotions in beverage consumption, regardless of the type of beverage. Thus, our findings are in agreement with previous studies.

When comparing the two formats of questionnaire items—words vs. full sentences—some minor differences, in terms of the number of significant emotion and wellness terms, were observed, depending on the measurement scale used. The number of significant terms for test 1 (rating-words) vs. test 2 (rating-sentences) was 13 vs. 14 for test 5 (RATA/scores-words) vs. test 6 (RATA/scores-sentences) was 15 vs. 14, for test 3 (CATA-words) vs. test 4 (CATA-sentences) was 12 vs. 13, and for test 5 (RATA/frequency-words) vs. test 6 (RATA/frequency-sentences) was 15 vs. 12. A greater difference was observed between words and sentences in the RATA frequency test format. However, overall, sentence format may not reduce ambiguity over the word-only format in the questionnaire. For terms unfamiliar to consumers (such as “polite”), including a context may help to increase understanding of the specified emotion or wellness state. However, the emotion and wellness terms used in the questionnaire in this study were pre-screened by consumers who regularly consume the herbal drink products. Consumers selected the terms that they were familiar with; therefore, a full sentence in the questionnaire may not provide much more clarity under the conditions of the current study.

The results from three types of measurement methods, including a rating scale, CATA, and RATA, were compared. The RATA data consisted of two types: RATA frequency and RATA score. Hence, the RATA scores were compared with rating scores, and RATA frequencies were compared with CATA frequencies.

**Figure 1:**
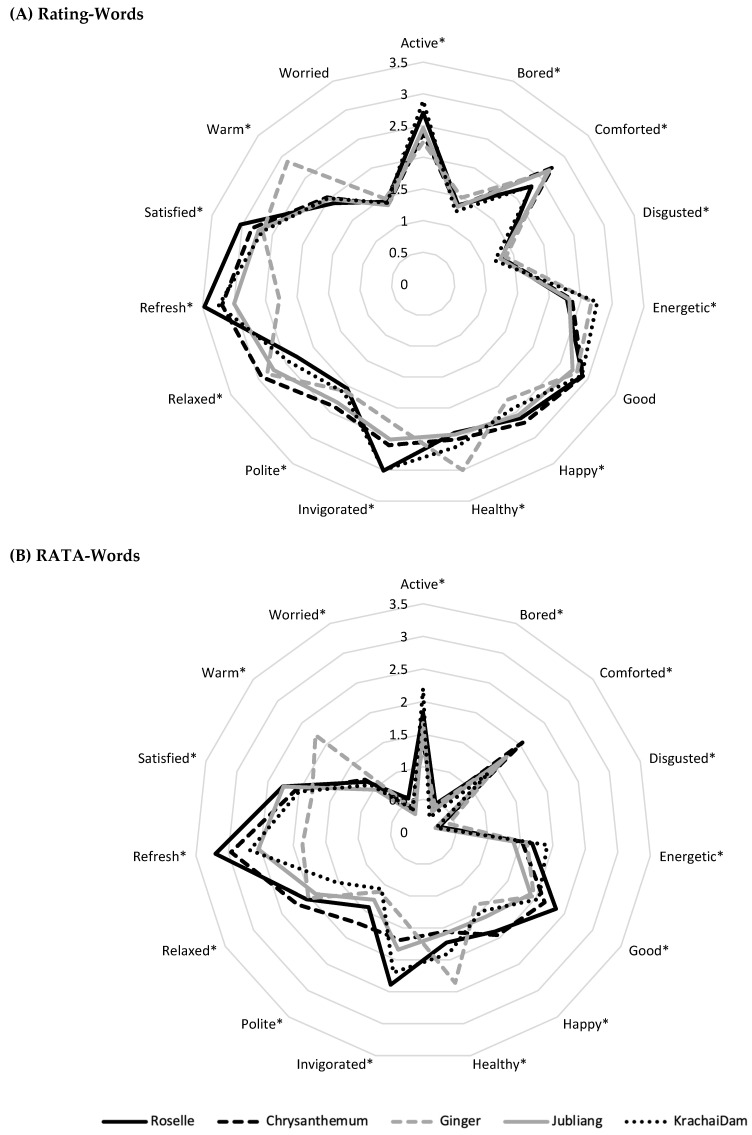
Emotion and wellness profiles of five herbal drinks from the (**A**) Rating-Words (score 1–5; *n* = 209) and (**B**) RATA-Words (score 0–5; *n* = 208) questionnaires. * indicates significant differences among the five herbal drinks (*p* < 0.05).

To compare rating scores (test 1) and RATA scores (test 5) from the word-format questionnaire, mean scores of emotion and wellness responses were plotted on a spider web, as shown in Figure 1. The overall profiles were visually similar, but the ratings from the rating scores were higher than those from the RATA scores. The rating-words profile (test 1) in Figure 1A reveals that significant product differences were observed for 13 terms (*p* < 0.05), except for “worried” (1.36–1.47 scores) and “good” (2.72–2.91 scores) terms (Table 6). Interestingly, the “refreshed” term showed higher scores of four herbal drinks (roselle, Jubliang, chrysanthemum, and Krachai Dam drink) (3.47, 3.00, 3.19, and 3.24, respectively) than other emotion and wellness terms. Meanwhile, the score of the “warm” term (2.88) was higher for ginger drink than other terms, with a maximum mean-difference value of 0.98. King et al. [29] suggested that a mean emotional difference of ≥ 0.2 units (on a 5-point scale) may be of practical value. For the rata-words profile (test 5) in Figure 1B, all 15 terms had significant differences among the five products. Therefore, using the RATA scale with words (Test 5) showed better discrimination ability than the rating scale with words (15 vs. 13) in terms of improving the number of significantly different terms.

**Figure 2:**
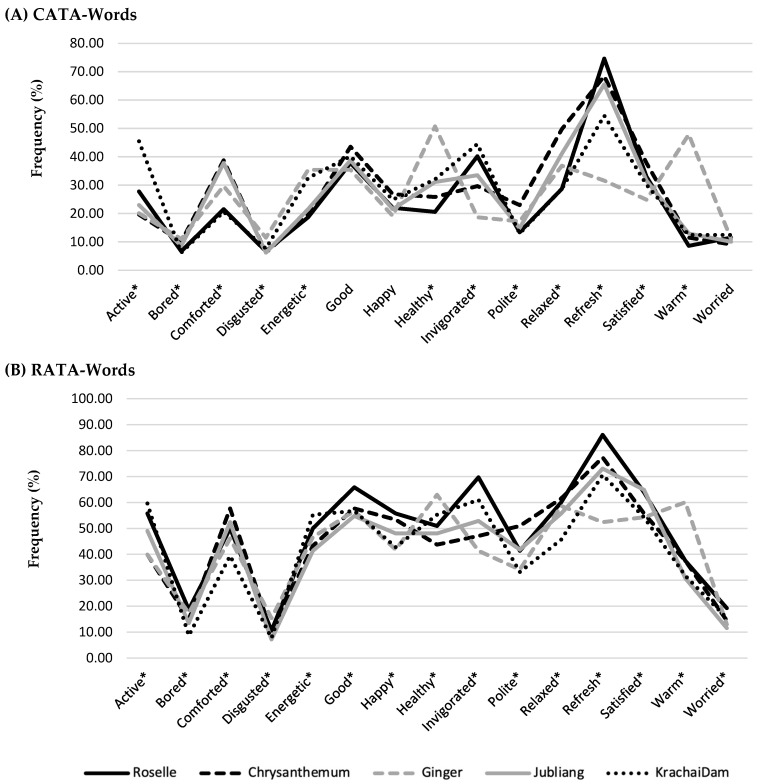
Emotion and wellness profiles of the five herbal drinks from the (**A**) CATA-Words (test 3; *n* = 209) and (**B**) RATA-Words (test 5; *n* = 208). * indicates significant differences among five herbal drinks (*p* < 0.05).

To compare the CATA frequency (test 3) and RATA frequency (test 5) from the word-format questionnaire, emotion and wellness responses, in terms of percentage of the frequency count, were plotted on a line chart, as shown in Figure 2. The two profiles were visually similar, but the frequency counts of the RATA-Words format was higher than those of the CATA-Words format. For CATA-Words in Figure 2A, the “refreshed” term was popular among consumers for roselle (74.64%), Jubliang (65.55%), chrysanthemum (68.42%), and Krachai Dam drink (54.55%), while “good”, “happy”, and “worried” terms were not significantly different among the five herbal drinks (*p* > 0.05; Table 7). For RATA-Words in Figure 2B, all 15 terms were significantly different among the five products. Thus, the RATA-Words show a slight improvement in the number of significantly different terms. Therefore, using the RATA scale with words (Test 5) showed slightly better discrimination ability than the CATA scale with words (15 vs. 12) in terms of increasing the number of significant terms.

Soares et al. [33] mentioned that the word-association (WA) method is used in consumer and marketing research to provide insights on perceptions and attitudes to achieve a deeper understanding of what consumers really think and feel about a product. Many researchers have studied the benefit of herbs, such as roselle, ginger and chrysanthemum, and found that they are a good source of vitamins and minerals and rich in phenolic compounds known to have free-radical-scavenging abilities [34,35,36,37,38]. In this study, consumers selected the “active” and “energetic” emotion terms, as well as the “invigorated”, “refreshed”, and “relaxed” wellness terms for these five herbal drink products. It is possible that some participants realized the important benefits of these herbal drink; thus, they chose those emotion and wellness terms based on their prior knowledge and consumption experiences. It is known that characteristics of an individual participant may affect the emotion profile of foods [2]; these characteristics include personal preference, past experience, frequency of consumption [30], and culture [39].

### 3.3. Discriminating Herbal Drinks Based on Emotion and Wellness Responses

To determine the overall product differences and which emotion and wellness attributes were mainly responsible for product discrimination, MANOVA and DA were performed (Table 8). When considering the MANOVA results of rating scores and RATA scores, significant differences were observed among the five herbal drinks in terms of emotion and wellness responses (*p* < 0.0001). Based on the first canonical dimension (Can 1), DA identified the “warm” emotion and the “refreshed” wellness terms as the two most discriminating terms among the herbal drinks based on questionnaire responses. However, one additional wellness term (“healthy”) was identified in Can 1 for the CATA frequency questionnaire. When considering Can 2, “active” and “comforted” terms significantly contributed to overall product differences. However, RATA scores identified one more wellness term (“relaxed”), while rating scores and CATA frequencies identified two more wellness terms (“relaxed” and “invigorated”). In summary, DA of CATA data revealed the greatest number of terms that discriminated the products, including two positive emotion terms (“warm” and “active”) and all five wellness terms (“refreshed”, “healthy”, “comforted”, “relaxed”, and “invigorated”) with greatest explained variance (93.6%). This finding (Table 8) is not in agreement with that previously reported based on the results from Table 6 and Table 7. One possible explanation is that the former data analysis took into account intercorrelations among emotion and wellness attributes, while the latter did not.

**Figure 3:**
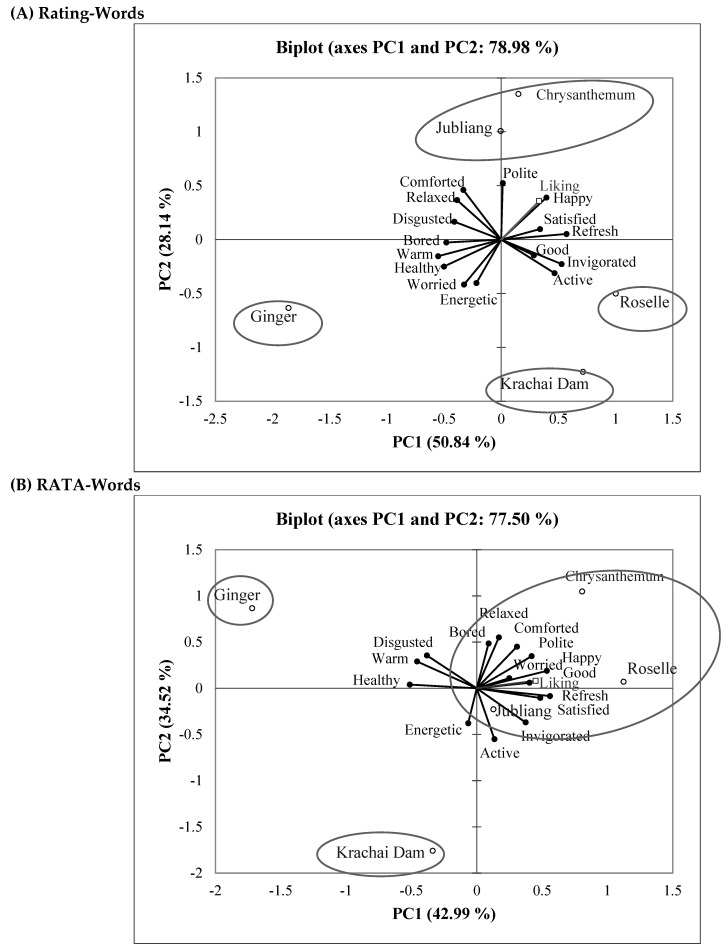
PCA biplots of emotion and wellness responses of the five herbal drinks in the (**A**) Rating-Words and (**B**) RATA-Words questionnaires. Symbols (○) and (●) indicate the position of herbal drinks and emotion-wellness terms, respectively.

PCA was performed to investigate the relationship between the five herbal drinks and emotion and wellness responses. PCA biplots of rating scores and RATA scores were compared (Figure 3A,B) with the first two PCs (PC1 and PC2), explaining 78.98% and 77.50% of total variance, respectively. For rating-words data, the PCA biplots identified four groups of herbal drinks: I-ginger drink, II-Krachai Dam drink, III-roselle drink, and IV-chrysanthemum-Jubliang drink (Figure 3A). Ginger drink and roselle drink corresponded with PC1 but in the opposite quadrant, while Krachai Dam drink and chrysanthemum-Jubliang drink corresponded with PC2 but in the opposite quadrant as well. Ginger drink correlated with “warm”, “healthy”, “disgusted”, and “bored”. Roselle drink correlated with “refreshed”, ”invigorated”, and “active”. Krachai Dam drink correlated with “energetic” and “worried”. Chrysanthemum and Jubliang drinks correlated with “polite” and “comforted”.

For RATA-words data, the PCA biplots revealed three groups of herbal drinks: I-ginger drink, II-Krachai Dam drink, and III-roselle, chrysanthemum, and Jubliang drinks (Figure 3B). Ginger drink and roselle drink corresponded with PC1, and Krachai Dam drink and Chrysanthemum drink corresponded with PC2, in opposite directions. Jubliang drink corresponded with PC3 (not shown in Figure 3), as it could not be explained using PC1 and PC2. Ginger drink correlated with “warm”, “healthy”, and “disgusted”. Roselle drink correlated with “refreshed”, ”invigorated”, “happy”, “satisfied”, and “polite”. Krachai Dam drink correlated with “energetic” and “active”. Chrysanthemum drink correlated with “polite”, “comforted”, and “bored”. Obviously, the “warm” emotion term and the “refreshed” wellness term were the most important terms differentiating the hot ginger drink from the cold herbal drinks (Table 8 and Figure 3).

**Figure 4:**
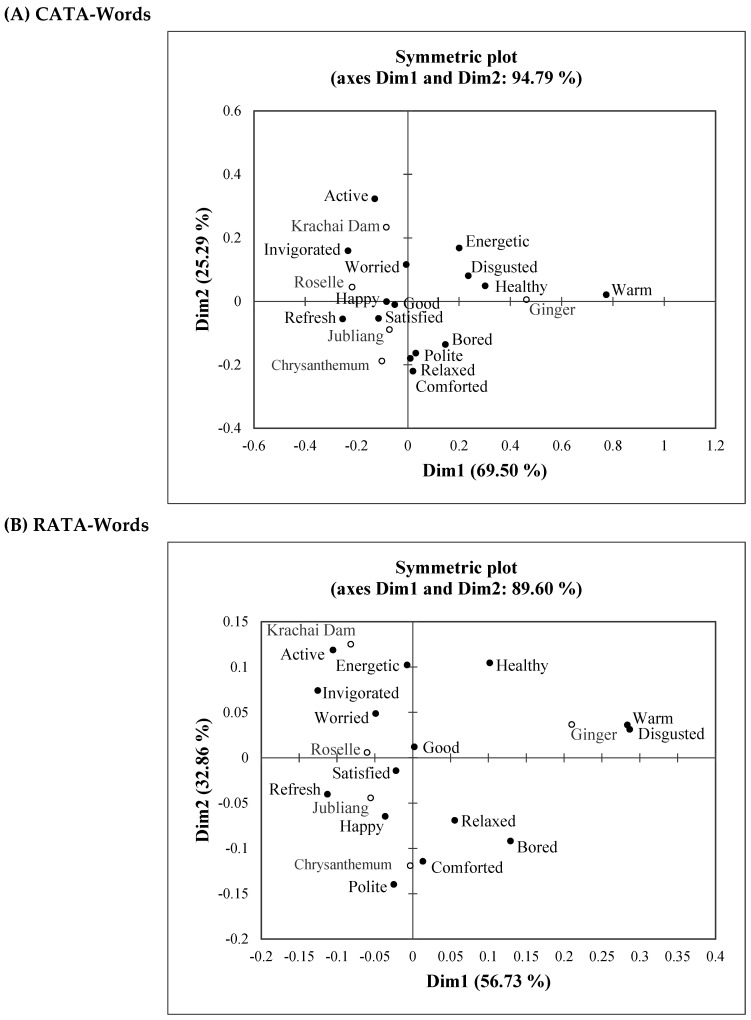
CA symmetric plots of emotion and wellness responses of the five herbal drinks in the (**A**) CATA-Words and (**B**) RATA-Words questionnaires. Symbols (○) and (●) indicate the position of herbal drinks and emotion and wellness terms, respectively.

The CA symmetric plots of CATA frequency and RATA frequency were compared (Figure 4A,B). The first two dimensions (Dim1 and Dim2) accounted for 94.79% and 89.60% of variance, respectively. CA configuration revealed four groups of herbal drinks: I-ginger drink, II-Krachai Dam drink, III-roselle drink, and IV-chrysanthemum-Jubliang drink. The ginger drink and the other four herbal drinks were clearly separated, on the opposite sides of Dim1 in both CA configurations. However, the CA configurations of CATA data vs. RATA data revealed some slightly different relationships between herbal drinks and emotion and wellness profiles. For CATA data (Figure 4A), ginger drink correlated with “warm”, “healthy”, and “disgusted”. Roselle drink correlated with “refreshed” and “invigorated”. Krachai Dam drink correlated with “active”. Chrysanthemum and Jubliang drinks correlated with “polite”, “relaxed”, and “comforted”. For RATA data (Figure 4B), ginger drink correlated with “warm” and “disgusted”. Roselle drink correlated with “refreshed” and “invigorated”. Krachai Dam drink correlated with “energetic” and “active”. Chrysanthemum and Jubliang drinks correlated with “polite”, “comforted”, “relaxed”, and ”happy”.

King et al. [31] evaluated the impact of rating scale and CATA on measurement of emotional responses. They found that the rating scale provided differentiation for more attributes at lower levels of emotional response, while CATA provided greater differentiation at higher levels of emotional frequency for a few select emotions. The rating scale was more sensitive than CATA, but both were found to be acceptable approaches, depending on the objective of the test [31]. In addition, Ng et al. [6] evaluated the effectiveness of the CATA approach compared to intensity scaling for the EsSense^®^ Profile. They reported that the qualitative nature of the data obtained from CATA limited the extent of statistical analysis, making it difficult to draw clear inferential conclusions from the data obtained with the EsSense Profile^®^. Therefore, using a combination of both approaches, RATA was proposed.

Meyners et al. [27] mentioned that compared to CATA questions, the use of a RATA variant was found to increase the number of attribute terms selected to describe samples and led to a slight increase in the percentage of terms for which significant differences among samples were identified. Jaeger et al. [40] compared the CATA and RATA question formats using an emoji questionnaire. They found that neither CATA nor RATA emoji-questions were regarded by consumers as difficult or tedious. Their recommendation for method selection was to use CATA emoji questions when samples have distinct emotional associations, whereas RATA seems to better discriminate between samples with more similar emotional profiles. However, in this study, the CA configurations of CATA data vs. RATA data revealed only slightly different relationships between herbal drinks and emotion and wellness profiles (Figure 4).

### 3.4. Overall Discussion

Overall, all types of data (rating, RATA, and CATA) showed similar results in terms of emotion and wellness profiles of the five herbal drinks studied. The “warm” and “healthy” terms were important and relevant to ginger drink, and the negative emotion term “disgusted” was elicited. “Refreshed” and “invigorated” were the important wellness terms for roselle drink. “Active”, “energetic”, and “worried” emotion terms were often elicited by Krachai Dam drink. Additionally, “comforted”, “relaxed”, and “bored” were significant terms for describing the chrysanthemum and Jubliang drinks. Although chrysanthemum drink had the highest overall liking scores (Table 5), some consumers expressed a “bored” emotion. It is a challenge of product developers to reduce the negative emotions associated with products. Elicited negative emotion terms should be carefully considered when developing or improving products. In the case of herbal drinks, ginger drink was associated with “disgusted” by some consumers, possibly because of its natural spicy flavor. Krachai Dam drink was associated with “worried” because it is not well-known by many Thai consumers and it normally imparts a pungent and sour flavor. “Bored” was elicited by chrysanthemum, possibly because it is a well-known traditional product that has not changed for a long time (i.e., not much product innovation applied to this drink).

When the three types of measuring methods—a rating scale, CATA question, and RATA question—were compared, they all produced similar emotion and wellness profiles, though there were advantages and limitations for each method. For a rating scale, the participants needed more time to complete the questionnaire than for other methods. However, a rating scale is appropriate for measuring intensity of attributes. The PCA and CA of rating scores were able to differentiate five herbal drinks into four groups with a higher percentage explained variance (78.98%) when compared with the RATA score (three groups; 77.50%). The CATA questionnaire was the most consumer-friendly method and was found to be appropriate for use for measurement of the presence or absence of the selected attributes. DA of CATA data identified the important terms discriminating the overall differences among the herbal tested drinks with the highest percentage of explained variance (93.6% total for Can1 and Can2). Additionally, CA of CATA data separated the five herbal drinks into four groups, similar to those of the rating data, with a higher percentage of explained variance (94.79%) when compared with the RATA frequency (four groups, 89.60%). However, the RATA score and RATA frequency from the RATA-Words question seemed to show better discrimination ability in term of improving the number of significant terms among the five herbal drinks.

## 4. Conclusions

In this study, we demonstrated the effects of two factors in designing a questionnaire for measurement of emotion and wellness responses elicited by herbal drinks consumed by Thai consumers. The first factor was questionnaire items in the form of words vs. full sentences. Results indicate that measuring emotion and wellness using full sentences did not provide a clear benefit over using words alone. When using familiar terms clearly understood by consumers, a full sentence in the questionnaire is not needed. For the second factor, three types of measuring methods, including a rating scale, CATA question, and RATA question were compared for their ability to discriminate among five herbal drinks. All three measuring methods produced similar emotion and wellness profiles. However, each method has advantages and limitations that researchers should carefully consider. Overall, this study provides some useful options in terms of questionnaire-item formats and measurement methods in the design of questionnaire to measuring consumers’ emotion and wellness responses to herbal drinks. The information elucidated in this study is applicable to other food products and beverages. In the future, the impact of health-benefit statements on consumer perception and purchase intent with regard to herbal drinks formulated in our lab will be assessed by product tasting and with an appropriate sample size representing the target population. We also plan to collaborate with a medical school for human clinical trials to determine the health benefits of these herbal drinks.

## Figures and Tables

**Table 1:** The description of five herbal drink samples.

Herbal Drinks	Main Ingredients	Serving Condition/Temperature
*Roselle drink* Ready to drink, 200 mL/box	Roselle extract 94%, sucrose 6%	Cold/10 °C
*Chrysanthemum drink* Ready to drink, 350 mL/bottle	Chrysanthemum extract 90.00%, fructose syrup 4.00%, sucrose 4.00%	Cold/10 °C
*Ginger drink* Instant powder ^1^, 18 g/sachet	Ginger extract 8.03%, sugar 10.47%, vitamin B1 0.0003%, vitamin B2 0.0003%, vitamin B6 0.0009%	Hot/70 °C
*Jubliang drink* Ready to drink, 300 mL/box	Jubliang extract 94.68%, fructose 4.0%, sugar 1.0%	Cold/10 °C
*Krachai Dam drink* Ready to drink, 90 mL/bottle	Krachai Dam extract 20%, fructose 18%, honey 1.5%, citric acid 0.5%, vitamin C 0.12%, vitamin A 0.012%, vitamin B6 0.004%	Cold/10 °C

^1^ ginger drink was prepared by diluting 18 g of instant ginger powder in 150 mL of hot water.

**Table 2:** Thai translation of emotion and wellness terms obtained from the EsSense Profile^®^ and the WellSense Profile^TM^.

EsSense Profile^®^ (39 Terms)					
Active	รู้สึกกระฉับกระเฉง	Glad	ยินดี/ดีใจ	Pleased	พึงพอใจ
Adventurous	ชอบผจญภัย	Good	ดี	Polite	สุภาพ
Affectionate	รักใคร่	Good-natured	มีใจเมตตา	Quiet	เงียบ
Aggressive	ก้าวร้าว	Guilty	รู้สึกผิด	Satisfied	พึงพอใจ
Bored	เบื่อ	Happy	มีความสุข	Secure	ปลอดภัย
Calm	สงบ	Interested	รู้สึกสนใจ	Steady	คงเส้นคงวา
Daring	กล้าหาญ	Joyful	ร่าเริง	Tame	จืดชืด/ไม่น่าสนใจ
Disgusted	น่ารังเกียจ/ขยะแขยง	Loving	รู้สึกรัก	Tender	นุ่มนวล
Eager	กระตือรือร้น	Merry	รู้สึกครึกครื้น	Understanding	มีความเข้าอกเข้าใจ
Energetic	รู้สึกมีพลัง	Mild	อ่อนโยน	Warm	อบอุ่น
Enthusiastic	กระตือรือร้น	Nostalgic	ระลึกถึงความหลัง	Whole	รู้สึกสมบูรณ์ครบถ้วน
Free	เป็นอิสระ	Peaceful	สงบ	Wild	คึกคะนอง
Friendly	เป็นมิตร	Pleasant	รู้สึกเพลิดเพลิน	Worried	รู้สึกกังวล
**WellSense Profile^TM^ (45 Terms)**					
Accepted	ได้รับการยอมรับ	Energetic	เต็มไปด้วยพลัง	Resilient	ยืดหยุ่น
Accomplished	บรรลุเป้าหมาย	Fatigued	เหนื่อยล้า	Rested	ได้พักผ่อน
Active	รู้สึกกระฉับกระเฉง	Focused	ตั้งใจจดจ่อ	Sad	เศร้า
Affectionate	รักใคร่	Friendly	เป็นมิตร	Satisfied	พึงพอใจ
Alert	ตื่นตัว	Fulfilled	รู้สึกได้รับการเติมเต็ม	Secure	ปลอดภัย
Approachable	เข้าถึงได้	Grateful	กตัญญูรู้คุณ	Sociable	ชอบเข้าสังคม
Attentive	เอาใจใส่	Happy	มีความสุข	Stimulated	ถูกกระตุ้น
Calm	สงบ	Healthy	มีสุขภาพดี	Stressed	เครียด
Comforted	รู้สึกสบาย	Invigorated	มีชีวิตชีวา	Supported	ได้รับการสนับสนุน
Compassionate	เห็นอกเห็นใจ	Joyful	ร่าเริง	Unfulfilled	ยังไม่ได้รับการเติมเต็ม
Concerned for others	มีความห่วงใยต่อผู้อื่น	Lonely	เหงา	Uninspired	ไม่มีแรงบันดาลใจ
Connected	ผูกพัน	Loved	รู้สึกได้รับความรัก	Uninterested	ไม่สนใจ
Creative	สร้างสรรค์	Peaceful	สงบ	Tense	เครียด
Curious	อยากรู้อยากเห็น	Refreshed	สดชื่น	Tired	เหนื่อยล้า
Disconnected	ไม่ผูกพัน	Relaxed	รู้สึกผ่อนคลาย	Whole	รู้สึกสมบูรณ์ครบถ้วน

**Table 3:** Selected emotional and wellness terms relevant to herbal drinks.

Term Categories	English	Thai
Positive emotional terms	Active	รู้สึกกระฉับกระเฉง
(7 terms)	Energetic	รู้สึกมีพลัง
	Good	รู้สึกดี
	Happy	รู้สึกมีความสุข
	Polite	รู้สึกสุภาพ
	Satisfied	รู้สึกพึงพอใจ
	Warm	รู้สึกอบอุ่น
Negative emotional terms	Bored	รู้สึกเบื่อ
(3 terms)	Disgusted	รู้สึกน่ารังเกียจ
	Worried	รู้สึกกังวล
Wellness terms	Comforted	รู้สึกสบาย
(5 terms)	Healthy	มีสุขภาพดี
	Invigorated	มีชีวิตชีวา
	Relaxed	รู้สึกผ่อนคลาย
	Refreshed	รู้สึกสดชื่น

**Table 4:** Demographic characteristics of participants for the six test designs.

Test Design	Test 1	Test 2	Test 3	Test 4	Test 5	Test 6	Total
Emotion and wellness items	Words	Sentences	Words	Sentences	Words	Sentences	
Measurement method	Rating	Rating	CATA	CATA	RATA	RATA	
Number of participants	209	208	209	207	208	211	1252
Gender (%) ^1^							
Male	49.8	49.5	49.3	49.3	49.0	48.8	49.3
Female	50.2	50.5	50.7	50.7	51.0	51.2	50.7
Age (%) ^1^							
18–21 years	24.9	24.5	24.4	26.6	25.2	27.5	25.6
22–40 years	24.9	26.0	25.8	24.2	26.5	24.6	25.2
41–59 years	25.4	25.5	25.8	25.1	24.4	24.2	25.1
≥60 years	24.8	24.0	24.0	24.1	23.9	23.7	24.1
Region of Thailand (%) ^1^							
Northern	20.1	20.2	21.1	18.8	20.7	19.4	20.1
Central	22.5	20.7	19.1	24.2	25.0	26.6	23.0
Northeastern	34.0	36.0	33.5	36.7	31.7	34.6	34.4
Southern	23.4	23.1	26.3	20.3	22.6	19.4	22.5

^1^ Percentage within each test design.

**Table 5:** The mean overall liking scores of the five herbal drinks in each test design.

Test Design ^1^	Roselle	Chrysanthemum	Ginger	Jubliang	Krachai Dam	F-Value ^3^	*p*-Value ^3^
1	7.11 ^a^ ± 1.33	7.32 ^a^ ± 1.35	6.48 ^c^ ± 2.03	6.81 ^b^ ± 1.28	6.65 ^bc^ ± 1.82	11.867	0.000
2	7.14 ^a^ ± 1.25	7.29 ^a^ ± 1.23	6.55 ^b^ ± 1.98	6.76 ^b^ ± 1.39	6.62 ^b^ ± 1.79	12.870	0.000
3	7.13 ^a^ ± 1.38	7.41 ^a^ ± 1.45	6.59 ^b^ ± 2.19	6.81 ^b^ ± 1.41	6.85 ^b^ ± 1.83	9.983	0.000
4	7.13 ^b^ ± 1.40	7.46 ^a^ ± 1.21	6.48 ^c^ ± 2.08	6.48 ^c^ ± 1.73	6.74 ^c^ ± 1.81	19.866	0.000
5	7.18 ^a^ ± 1.33	7.35 ^a^ ± 1.42	6.81 ^b^ ± 2.04	6.87 ^b^ ± 1.51	7.06 ^ab^ ± 1.74	4.841	0.001
6	7.23 ^a^ ± 1.28	7.49 ^a^ ± 1.22	6.86 ^b^ ± 1.96	6.79 ^b^ ± 1.59	6.64 ^c^ ± 2.01	13.335	0.000
**F-value** ^2^	0.179	0.753	1.439	1.478	1.184	-	-
***p*-value** ^2^	0.970	0.584	0.208	0.195	0.315	-	-

Mean ± SD from 1252 consumer responses based on a 9-point hedonic scale. ^a–c^ Different letters in the same row indicate significant differences across samples according to Duncan’s multiple range test (*p* < 0.05). ^1^ Test 1 rating, words; Test 2 rating, sentences; Test 3 CATA, words; Test 4 CATA, sentences; Test 5 RATA, words; Test 6 RATA, sentences. ^2^ F-value and *p*-value in ANOVA were used to assess whether there is a difference among the six test designs. ^3^ F-value and *p*-value in ANOVA were used to assess whether there is a difference among the five herbal drinks.

**Table 6:** Mean ± standard deviations of overall liking ^1^ and emotion/wellness responses elicited by five herbal drinks in rating ^2^ and RATA ^3^ questionnaires.

Attributes	Test Samples					*p*-Value
	R	C	G	J	K	
**Test design 1: Rating-Words** (*n* = 209)						
**Overall liking**	7.11 ± 1.33 a	7.32 ± 1.35 a	6.48 ± 2.03 c	6.81 ± 1.28 b	6.65 ± 1.82 bc	<0.001
**Emotion**						
Active	2.70 ± 1.29 a	2.37 ± 1.33 bc	2.24 ± 1.36 c	2.46 ± 1.33 b	2.89 ± 1.38 a	<0.001
Energetic	2.28 ± 1.32 b	2.36 ± 1.35 b	2.67 ± 1.34 a	2.32 ± 1.32 b	2.76 ± 1.36 a	<0.001
Good	2.90 ± 1.31	2.91 ± 1.37	2.80 ± 1.41	2.72 ± 1.33	2.89 ± 1.34	0.311
Happy	2.62 ± 1.31 a	2.71 ± 1.32 a	2.26 ± 1.34 c	2.56 ± 1.26 ab	2.42 ± 1.37 bc	<0.001
Polite	2.04 ± 1.26 b	2.36 ± 1.29 a	2.09 ± 1.28 b	2.31 ± 1.14 a	2.12 ± 1.33 b	<0.001
Satisfied	3.03 ± 1.25 a	2.84 ± 1.34 ab	2.66 ± 1.33 b	2.73 ± 1.23 b	2.67 ± 1.44 b	0.003
Warm	1.90 ± 1.15 b	2.04 ± 1.31 b	2.88 ± 1.34 a	2.00 ± 1.15 b	2.02 ± 1.26 b	<0.001
Bored	1.36 ± 0.81 b	1.33 ± 0.74 bc	1.49 ± 0.88 a	1.33 ± 0.77 bc	1.25 ± 0.66 c	<0.001
Disgusted	1.30 ± 0.81 ab	1.31 ± 0.80 a	1.37 ± 0.83 a	1.28 ± 0.71 ab	1.19 ± 0.58 b	0.020
Worried	1.42 ± 1.02	1.36 ± 0.86	1.47 ± 0.90	1.36 ± 0.78	1.40 ± 0.79	0.403
**Wellness**						
Comforted	2.30 ± 1.31 b	2.74 ± 1.33 a	2.63 ± 1.40 a	2.68 ± 1.22 a	2.32 ± 1.33 b	<0.001
Healthy	2.40 ± 1.26 c	2.51 ± 1.33 bc	3.00 ± 1.32 a	2.43 ± 1.27 bc	2.62 ± 1.35 b	<0.001
Invigorated	3.01 ± 1.30 a	2.60 ± 1.43 b	2.26 ± 1.35 c	2.51 ± 1.33 b	3.00 ± 1.25 a	<0.001
Relaxed	2.30 ± 1.28 c	2.94 ± 1.21 a	2.84 ± 1.30 ab	2.72 ± 1.27 b	2.45 ± 1.38 c	<0.001
Refreshed	3.47 ± 1.18 a	3.19 ± 1.31 bc	2.28 ± 1.32 d	3.00 ± 1.19 c	3.24 ± 1.15 b	<0.001
**Test design 2: Rating-Sentences** (*n* = 208)						
**Overall liking**	7.14 ± 1.25 a	7.29 ± 1.23 a	6.55 ± 1.98 b	6.76 ± 1.39 b	6.62 ± 1.79 b	<0.001
**Emotion**						
Active	2.58 ± 1.24 ab	2.41 ± 1.26 b	2.18 ± 1.24 c	2.43 ± 1.38 ab	2.61 ± 1.28 a	<0.001
Energetic	2.32 ± 1.16 b	2.28 ± 1.20 b	2.38 ± 1.31 b	2.22 ± 1.15 b	2.73 ± 1.27 a	<0.001
Good	2.97 ± 1.24 a	2.98 ± 1.35 a	2.57 ± 1.33 c	2.75 ± 1.22 bc	2.81 ± 1.34 ab	<0.001
Happy	2.63 ± 1.24 b	2.85 ± 1.26 a	2.35 ± 1.27 c	2.55 ± 1.30 b	2.51 ± 1.34 bc	<0.001
Polite	2.08 ± 1.17 c	2.49 ± 1.31 a	2.03 ± 1.15 c	2.27 ± 1.18 b	2.00 ± 1.17 c	<0.001
Satisfied	2.94 ± 1.26 a	2.89 ± 1.26 a	2.49 ± 1.22 b	2.78 ± 1.26 a	2.74 ± 1.38 a	<0.001
Warm	2.11 ± 1.19 b	2.19 ± 1.23 b	2.81 ± 1.30 a	2.08 ± 1.16 b	2.08 ± 1.20 b	<0.001
Bored	1.47 ± 0.94 a	1.29 ± 0.68 b	1.34 ± 0.71 ab	1.43 ± 0.83 a	1.43 ± 0.87 a	0.034
Disgusted	1.31 ± 0.74 bc	1.24 ± 0.62 c	1.42 ± 0.85 a	1.27 ± 0.65 c	1.39 ± 0.85 ab	0.002
Worried	1.40 ± 0.83	1.37 ± 0.81	1.38 ± 0.78	1.43 ± 0.83	1.50 ± 0.86	0.119
**Wellness**						
Comforted	2.54 ± 1.27 c	3.07 ± 1.28 a	2.54 ± 1.31 c	2.75 ± 1.17 b	2.43 ± 1.37 c	<0.001
Healthy	2.53 ± 1.26 b	2.56 ± 1.29 b	2.99 ± 1.26 a	2.47 ± 1.30 b	2.63 ± 1.26 b	<0.001
Invigorated	3.08 ± 1.15 a	2.88 ± 1.32 b	2.27 ± 1.23 c	2.70 ± 1.25 b	2.87 ± 1.28 b	<0.001
Relaxed	2.46 ± 1.26 b	2.79 ± 1.27 a	2.56 ± 1.28 b	2.51 ± 1.29 b	2.25 ± 1.30 c	<0.001
Refreshed	3.32 ± 1.12 a	3.52 ± 1.16 a	2.45 ± 1.27 c	3.03 ± 1.19 b	3.06 ± 1.19 b	<0.001
**Test design 5: RATA-Words** (*n* = 208)						
**Overall liking**	7.18 ± 1.33 a	7.35 ± 1.42 a	6.81 ± 2.04 b	6.87 ± 1.51 b	7.06 ± 1.74 ab	<0.001
**Emotion**						
Active	1.83 ± 1.73 b	1.40 ± 1.81 c	1.33 ± 1.73 c	1.67 ± 1.85 b	2.20 ± 1.97 a	<0.001
Energetic	1.68 ± 1.79 ab	1.53 ± 1.85 bc	1.60 ± 1.82 bc	1.39 ± 1.75 c	1.91 ± 1.89 a	0.001
Good	2.35 ± 1.80 a	2.15 ± 1.96 ab	1.96 ± 1.86 c	1.90 ± 1.83 c	2.05 ± 1.94 ab	0.023
Happy	1.88 ± 1.77 a	1.95 ± 1.92 a	1.37 ± 1.71 b	1.61 ± 1.78 b	1.50 ± 1.83 b	<0.001
Polite	1.42 ± 1.79 b	1.72 ± 1.79 a	1.13 ± 1.63 c	1.28 ± 1.64 bc	1.08 ± 1.62 c	<0.001
Satisfied	2.26 ± 1.80 a	2.06 ± 1.92 ab	1.79 ± 1.76 b	2.25 ± 1.79 a	2.00 ± 1.96 ab	0.008
Warm	1.15 ± 1.56 b	1.20 ± 1.66 b	2.22 ± 1.98 a	0.97 ± 1.52 b	1.08 ± 1.66 b	<0.001
Bored	0.47 ± 1.01 a	0.41 ± 1.04 a	0.44 ± 0.99 a	0.34 ± 0.89 ab	0.24 ± 0.80 b	0.004
Disgusted	0.26 ± 0.81 b	0.26 ± 0.86 b	0.41 ± 1.01 a	0.20 ± 0.75 b	0.21 ± 0.76 b	0.009
Worried	0.56 ± 1.22 a	0.41 ± 1.05 b	0.40 ± 1.09 b	0.30 ± 0.87 b	0.38 ± 0.95 b	0.014
**Wellness**						
Comforted	1.71 ± 1.80 b	2.05 ± 1.87 a	1.59 ± 1.82 bc	1.72 ± 1.74 b	1.35 ± 1.79 c	<0.001
Healthy	1.73 ± 1.78 bc	1.56 ± 1.86 c	2.36 ± 1.94 a	1.59 ± 1.78 c	1.90 ± 1.84 b	<0.001
Invigorated	2.39 ± 1.71 a	1.69 ± 1.89 b	1.38 ± 1.74 c	1.84 ± 1.86 b	2.20 ± 1.90 a	<0.001
Relaxed	2.06 ± 1.79 ab	2.22 ± 1.85 a	2.03 ± 1.85 ab	1.90 ± 1.82 b	1.54 ± 1.81 c	<0.001
Refreshed	3.20 ± 1.46 a	2.96 ± 1.74 ab	1.86 ± 1.90 d	2.54 ± 1.71 c	2.67 ± 1.86 bc	<0.001
**Test design 6: RATA-Sentences** (*n* = 211)						
**Overall liking**	7.23 ± 1.28 a	7.49 ± 1.22 a	6.86 ± 1.96 b	6.79 ± 1.59 b	6.64 ± 2.01 b	<0.001
**Emotion**						
Active	1.97 ± 1.76 a	1.71 ± 1.79 b	1.49 ± 1.68 b	1.54 ± 1.67 b	1.93 ± 1.76 a	<0.001
Energetic	1.78 ± 1.69 b	1.47 ± 1.68 c	1.68 ± 1.63 bc	1.51 ± 1.68 c	2.07 ± 1.70 a	<0.001
Good	2.56 ± 1.70 a	2.43 ± 1.74 ab	2.40 ± 1.71 ab	2.26 ± 1.71 c	2.17 ± 1.76 c	0.039
Happy	2.06 ± 1.72 a	2.14 ± 1.81 a	1.65 ± 1.74 b	2.08 ± 1.69 a	1.41 ± 1.67 c	<0.001
Polite	1.36 ± 1.59 b	1.62 ± 1.63 a	1.29 ± 1.62 b	1.48 ± 1.61 ab	1.31 ± 1.70 b	0.008
Satisfied	2.57 ± 1.69 a	2.42 ± 1.77 a	2.28 ± 1.72 ab	2.37 ± 1.65 a	2.05 ± 1.84 b	0.007
Warm	1.40 ± 1.66 b	1.31 ± 1.64 b	2.39 ± 1.75 a	1.27 ± 1.65 b	1.27 ± 1.62 b	<0.001
Bored	0.47 ± 1.04 ab	0.34 ± 0.91 b	0.48 ± 1.14 ab	0.38 ± 0.95 b	0.54 ± 1.18 a	0.039
Disgusted	0.35 ± 0.96	0.23 ± 0.79	0.39 ± 1.10	0.25 ± 0.79	0.32 ± 0.93	0.089
Worried	0.34 ± 0.90 c	0.36 ± 1.03 c	0.44 ± 1.16 bc	0.54 ± 1.15 ab	0.64 ± 1.18 a	0.001
**Wellness**						
Comforted	1.94 ± 1.76 b	2.13 ± 1.79 ab	1.98 ± 1.74 b	2.27 ± 1.66 a	1.61 ± 1.72 c	<0.001
Healthy	1.86 ± 1.76 c	1.74 ± 1.78 c	2.64 ± 1.71 a	1.72 ± 1.78 c	2.19 ± 1.80 b	<0.001
Invigorated	2.52 ± 1.67 a	1.88 ± 1.78 b	1.58 ± 1.71 c	2.02 ± 1.73 b	2.36 ± 1.74 a	<0.001
Relaxed	1.80 ± 1.73 bc	2.14 ± 1.73 a	2.00 ± 1.61 ab	1.75 ± 1.65 c	1.50 ± 1.71 d	<0.001
Refreshed	3.09 ± 1.44 a	3.07 ± 1.52 a	1.93 ± 1.81 c	2.79 ± 1.36 b	2.90 ± 1.55 ab	<0.001

^1^ Mean ± SD of overall liking scores based on a 9-points hedonic scale (1 = extremely dislike to 5 = extremely like). ^2^ Mean ± SD of rating scores based on a 5-points scale (1 = not at all to 5 = extremely). ^3^ Mean ± SD of RATA rating scores based on a 5-points scale (1 = slightly to 5 = extremely). a–d Mean values in the same row followed by different letters are significantly different (*p* < 0.05). Note: R = Roselle drink; C = Chrysanthemum drink; G = Ginger drink; J = Jubliang drink; K = Krachai Dam drink.

**Table 7:** Mean ± standard deviations of overall liking ^1^ and frequency counts of emotion/wellness responses elicited by five herbal drinks in CATA ^2^ and RATA ^2^ questionnaires.

Attributes	Test Samples					Cochran’s Q	*p*-Value
	R	C	G	J	K		
**Test design 3: CATA-Words** (*n* = 209)							
**Overall liking**	7.13 ± 1.38 a	7.41 ± 1.45 a	6.59 ± 2.19 b	6.81 ± 1.41 b	6.85 ± 1.83 b		<0.001
**Emotion**							
Active	58	41	42	48	95	68.962	<0.001
Energetic	39	41	74	45	68	45.305	<0.001
Good	79	91	74	81	84	4.603	0.331
Happy	46	56	40	45	52	8.082	0.089
Polite	28	48	36	31	27	16.994	0.002
Satisfied	69	79	52	66	63	13.338	0.010
Warm	18	24	100	27	26	222.749	<0.001
Bored	14	21	23	18	13	14.667	0.005
Disgusted	14	13	24	13	16	21.500	<0.001
Worried	24	19	23	21	26	3.476	0.482
**Wellness**							
Comforted	45	81	62	79	43	42.904	<0.001
Healthy	43	54	106	65	67	71.609	<0.001
Invigorated	84	62	39	70	93	52.529	<0.001
Relaxed	60	104	77	86	60	40.209	<0.001
Refreshed	156	143	66	137	114	119.685	<0.001
**Test design 4: CATA-Sentences** (*n* = 207)							
**Overall liking**	7.13 ± 1.40 b	7.46 ± 1.21 a	6.48 ± 2.08 c	6.48 ± 1.73 c	6.74 ± 1.81 c		<0.001
**Emotion**							
Active	53	47	40	42	90	68.784	<0.001
Energetic	44	33	64	30	72	65.502	<0.001
Good	85	108	79	89	81	17.227	0.002
Happy	64	82	46	53	53	36.553	<0.001
Polite	20	47	32	41	16	45.342	<0.001
Satisfied	72	73	49	70	67	14.916	0.005
Warm	22	35	82	41	33	106.995	<0.001
Bored	18	19	22	24	21	4.750	0.314
Disgusted	17	16	25	23	22	14.571	0.006
Worried	15	18	20	21	22	5.500	0.240
**Wellness**							
Comforted	75	102	76	95	66	27.069	<0.001
Healthy	61	115	69	63	68	65.954	<0.001
Invigorated	81	77	48	79	105	46.724	<0.001
Relaxed	71	77	77	58	47	24.281	<0.001
Refreshed	160	141	77	123	124	89.387	<0.001
**Test design 5: RATA-Words** (*n* = 208)							
**Overall liking**	7.18 ± 1.33 a	7.35 ± 1.42 a	6.81 ± 2.04 b	6.87 ± 1.51 b	7.06 ± 1.74 ab		<0.001
**Emotion**							
Active	116	83	83	102	124	52.089	<0.001
Energetic	104	90	97	86	115	21.243	<0.001
Good	137	120	118	114	118	10.919	0.027
Happy	116	111	87	100	89	25.585	<0.001
Polite	86	106	71	87	69	39.768	<0.001
Satisfied	133	116	113	135	113	15.948	0.003
Warm	78	78	125	64	66	94.646	<0.001
Bored	39	31	36	28	18	21.387	<0.001
Disgusted	22	20	32	15	16	17.864	0.001
Worried	40	30	27	24	32	10.590	0.032
**Wellness**							
Comforted	104	120	96	109	82	30.556	<0.001
Healthy	106	91	131	100	115	34.691	<0.001
Invigorated	145	98	86	110	127	69.516	<0.001
Relaxed	125	128	122	116	95	21.713	<0.001
Refreshed	179	161	109	152	147	81.199	<0.001
**Test design 6: RATA-Sentences** (*n* = 211)							
**Overall liking**	7.23 ± 1.28 a	7.49 ± 1.22 a	6.86 ± 1.96 b	6.79 ± 1.59 b	6.64 ± 2.01 b		<0.001
**Emotion**							
Active	127	109	98	103	126	34.400	<0.001
Energetic	118	99	116	101	137	41.902	<0.001
Good	157	149	151	144	139	6.643	0.156
Happy	133	130	108	135	95	56.124	<0.001
Polite	96	112	88	105	88	22.218	<0.001
Satisfied	158	147	144	151	125	20.266	<0.001
Warm	95	90	147	84	89	121.892	<0.001
Bored	38	27	35	31	39	7.353	0.118
Disgusted	26	18	26	20	24	4.935	0.294
Worried	28	26	31	42	53	28.398	<0.001
**Wellness**							
Comforted	123	132	127	148	108	36.416	<0.001
Healthy	119	113	158	107	137	64.692	<0.001
Invigorated	158	120	104	129	147	65.381	<0.001
Relaxed	116	137	136	118	99	39.635	<0.001
Refreshed	184	180	122	181	175	96.239	<0.001

^1^ Mean ± SD of overall liking scores based on a 9-point hedonic scale (1 = extremely dislike, 5 = extremely like). ^2^ Frequency counts of selected emotion/wellness terms were analyzed using Cochran’s Q test. a–c Mean values in the same row followed by different letters are significantly different (*p* < 0.05). Note: R = roselle drink; C = chrysanthemum drink; G = ginger drink; J = Jubliang drink; K = Krachai Dam drink.

**Table 8:** Canonical structure (r’s) ^1^ describing group differences among the five herbal drinks based on emotion and wellness responses obtained from the rating, CATA, and RATA questionnaires.

Responses	Rating Scores ^2^		RATA Scores ^2^		CATA Frequencies ^2^		RATA Frequencies ^2^	
	Can 1	Can 2	Can 1	Can 2	Can 1	Can 2	Can 1	Can 2
**Emotion**								
Active	0.181	**−0.319**	0.148	**−0.402**	0.109	−0.531	0.177	**−0.349**
Energetic	−0.142	−0.232	−0.001	−0.191	−0.210	−0.234	0.003	−0.224
Good	0.037	−0.039	0.079	0.025	0.059	0.044	0.055	−0.011
Happy	0.162	0.159	0.174	0.173	0.069	0.014	0.159	0.147
Polite	0.032	0.228	0.134	0.287	−0.017	0.210	0.139	0.284
Satisfied	0.117	0.028	0.148	0.008	0.120	0.111	0.114	0.035
Warm	**−0.457**	−0.020	**−0.465**	0.137	**−0.648**	0.036	**−0.461**	0.114
Bored	−0.122	0.041	−0.027	0.161	−0.071	0.127	−0.043	0.140
Disgusted	−0.072	0.092	−0.139	0.071	−0.115	−0.044	−0.165	0.053
Worried	−0.049	−0.060	0.030	0.010	−0.005	−0.097	0.061	−0.053
**Wellness**								
Comforted	−0.091	**0.316**	0.075	**0.316**	−0.013	**0.409**	0.080	**0.302**
Healthy	−0.259	−0.088	−0.267	−0.112	**−0.348**	−0.045	−0.222	−0.177
Invigorated	0.282	**−0.315**	0.264	−0.281	0.248	**−0.303**	0.293	−0.284
Relaxed	−0.166	**0.355**	−0.003	**0.313**	−0.004	**0.399**	−0.016	0.259
Refreshed	**0.525**	−0.110	**0.441**	0.051	**0.493**	0.192	**0.485**	0.059
% Cumulative variance explained	62.5	89.0	61.7	90.7	68.5	93.6	54.4	87.5
MANOVA; Wilks’ Lambda *p*-value	<0.0001		<0.0001					

^1^ Based on the pooled within-group variance with *p* < 0.0001 for rating score, RATA score, CATA frequency, and RATA frequency. Can1 and Can2 refer to the first and second canonical discrimination function, respectively. ^2^ Rating scores from Rating-Words question, RATA scores; RATA frequencies from RATA-Words question; CATA frequencies from CATA-Words question.

## Data Availability

The data presented in this study are available in the manuscript.
